# Prognostic impact of myelodysplasia-related gene mutations in *FLT3*-ITD-mutated acute myeloid leukemia

**DOI:** 10.1038/s41375-026-02874-w

**Published:** 2026-02-09

**Authors:** Rabea Mecklenbrauck, Angela Villaverde Ramiro, Eric Sträng, Razif Gabdoulline, Javier Martinez Elicegui, Marta Sobas, Lisa Pleyer, Amin Turki, Maria Teresa Voso, Axel Benner, Alberto Hernández-Sánchez, Jesse M. Tettero, Laura Tur Gimenez, Klaus H. Metzeler, Guadalupe Oñate, Sören Lehmann, Brian JP Huntly, Ian Thomas, Felicitas R. Thol, Florian H. Heidel, Peter JM Valk, Konstanze Döhner, Torsten Haferlach, Kenneth I. Mills, Hartmut Döhner, Gastone Castellani, Gert J. Ossenkoppele, Jesus María Hernández-Rivas, Lars Bullinger, Michael Heuser

**Affiliations:** 1 Department of Hematology, Hemostasis, Oncology and Stem Cell Transplantation, Hannover Medical School, Hannover, Germany; 2 Weatherall Institute of Molecular Medicine, University of Oxford, Oxford, United Kingdom; 3 Institute of Biomedical Research of Salamanca (IBSAL), Salamanca, Spain; 4 Fundación HARMONY Alliance Foundation (HAF), Salamanca, Spain; 5 Charité Universitätsmedizin Berlin, Berlin, Germany; 6 Department of Hematology, Collegium Medicum in Bydgoszcz, Nicolaus Copernicus University in Toruń, Bydgoszcz, Poland; 7 Paracelsus Medical University Hospital Salzburg, Salzburg, Austria; 8 Department of Hematology and Oncology, Marienhospital University Hospital, Ruhr-University Bochum, Bochum, Germany; 9 University of Rome Tor Vergata, Rome, Italy; 10 Division of Biostatistics, German Cancer Research Center (DKFZ), Heidelberg, Germany; 11 Hematology Department, University Hospital of Salamanca, Salamanca, Spain; 12 Amsterdam University Medical Centers, location VUmc, Amsterdam, the Netherlands; 13 GMV Innovating Solutions, Valencia, Spain; 14 Department of Hematology, Cell Therapy, Hemostaseology and Infectious Diseases, University of Leipzig, Leipzig, Germany; 15 Hospital Santa Creu i Sant Pau, Barcelona, Spain; 16 Karolinska Institutet, Stockholm, Sweden; 17 Wellcome-MRC Cambridge Stem Cell Institute, University of Cambridge, Cambridge, United Kingdom; 18 Centre for Trials Research, Cardiff University, Cardiff, United Kingdom; 19 Erasmus University Medical Center Cancer Institute, Rotterdam, the Netherlands; 20 Department of Internal Medicine III, University Hospital of Ulm, Ulm, Germany; 21 MLL Munich Leukemia Laboratory, Munich, Germany; 22 Queens University Belfast, Belfast, United Kingdom; 23 Department of Medical and Surgical Sciences (DIMEC), University of Bologna, Bologna, Italy; 24 University Hospital Halle (Saale), Department of Internal Medicine IV, Martin-Luther-University Halle-Wittenberg, Halle, Germany

**Keywords:** Acute myeloid leukaemia, Genetics research

## Abstract

The inclusion of nine myelodysplasia-related gene (MRG) mutations (*ASXL1, BCOR, EZH2, RUNX1, SF3B1, SRSF2, STAG2, U2AF1, ZRSR2*) as adverse risk factors in the ELN risk classification has reshaped classification in acute myeloid leukemia (AML). AML with *FLT3*-ITD mutations and co-occurring MRG alterations is now classified to the ELN adverse risk group although supporting evidence remains limited. Among 4,078 patients with AML with available molecular information included in the HARMONY platform, 862 harbored *FLT3*-ITD mutations and underwent intensive chemotherapy. Of these, 171 (20%) exhibited co-occurring MRG mutations at diagnosis. In this cohort, MRGs were not independently associated with relapse-free survival (RFS) or overall survival (OS). In the *FLT3*-ITD/*NPM1* co-mutated subgroup, MRG mutations were rare (9%) and showed no prognostic impact. Conversely, in *FLT3-*ITD*/NPM1* wildtype AML, MRG mutations were predictive of shorter RFS (HR 1.37, 95%CI 1.01 – 1.88, *p* = 0.046) and OS (HR 1.34, 95%CI 1.02–1.74, *p* = 0.032) in multivariable analysis with survival times comparable to the ELN adverse risk category. The allelic ratio of *FLT3*-ITD did not further stratify OS and RFS in this subgroup. These findings suggest that the prognostic relevance of MRG mutations in *FLT3*-ITD AML is modulated by *NPM1* co-mutational status and mirror findings in AML lacking *FLT3*-ITD.

## Introduction

Acute myeloid leukemia (AML) is a heterogeneous malignancy for which prognosis is strongly influenced by the presence of specific gene mutations. The 2022 International Consensus Classification (ICC) introduced a novel genetically defined subgroup of AML characterized by myelodysplasia-related gene (MRG) mutations [1]. This subgroup is defined by the presence of at least one mutation in any of the following nine genes: *ASXL1, BCOR, EZH2, RUNX1, SF3B1, SRSF2, STAG2, U2AF1*, or *ZRSR2* [1]. A similar change was suggested in the most recent WHO classification although this definition excluded *RUNX1* [2]. These mutations are generally associated with poor prognosis and, in the absence of co-occurring favorable genetic alterations, are classified as adverse risk according to the 2022 European LeukemiaNet (ELN) classification [3, 4].

The prognostic relevance of these mutations remains a subject of ongoing evaluation. Emerging evidence suggests that only *RUNX1* and *ASXL1* mutations are consistently associated with adverse outcomes while the remaining MRG mutations may confer a prognosis more comparable to the ELN-defined intermediate risk category [5–7]. We and others have demonstrated that the prognostic impact of MRG mutations may be influenced by their variant allele frequency (VAF) while additional studies have reported that only the presence of multiple MRG mutations within a single patient is associated with reduced overall survival (OS) [5, 8–11]. Furthermore, the impact of co-occurring mutations on the prognostic significance of MRG mutations remains uncertain. Several studies have investigated cases in which MRG mutations coincide with genetic or cytogenetic alterations typically associated with favorable prognosis, particularly *NPM1* mutations. Some studies support the ELN recommendation to classify such patients as favorable risk [12], while others report persistent adverse outcome with MRG mutations in *NPM1*-mutated AML [13]. Notably, a recent study suggested that negative measurable residual disease (MRD) after two cycles of chemotherapy may mitigate the negative prognostic effect of MRG mutations in this context [14].

Apart from *NPM1, FLT3* is one of the most frequently mutated genes in AML [15]. *FLT3* mutations frequently occur as internal tandem duplications (ITDs) or missense mutations in the tyrosine kinase domain (TKD). ITD mutations, in particular, have been associated with elevated white blood cell (WBC) counts, a high percentage of bone marrow blasts, and reduced OS [16, 17]. Patients with internal tandem duplications of *FLT3* and a high allelic ratio (AR) ≥ 0.5 in the absence of *NPM1* mutations were previously classified as adverse risk [18]. However, the 2022 ELN risk classification now categorizes all *FLT3*-ITD positive patients as intermediate risk, regardless of AR or co-occurring *NPM1* mutations [4]. This revision, among other reasons, takes into account the significant survival improvement of *FLT3*-ITD positive patients following the introduction of FLT3 inhibitors (FLT3i) [19–22]. Nonetheless, a Spanish cohort study implicated that the impact of AR remained evident in *FLT3*-ITD positive patients treated with FLT3i [23]. It has also been suggested that the prognostic relevance of AR is less pronounced following allogeneic hematopoietic cell transplantation (alloHCT) [6].

To date, the prognostic impact of MRG mutations co-occurring with *FLT3* mutations has not yet been systematically evaluated, and these patients are currently classified as having adverse risk disease. In this study, we identified *FLT3*-ITD positive patients who underwent intensive treatment within the HARMONY Platform, with the aim to assess the specific prognostic impact of MRG mutations in this genetic context.

## Patients, materials, and methods

### Patients

At the time of data cutoff (01 Mar 2025) 34 770 AML patients were included in the HARMONY Platform. We selected patients aged ≥18 years, who had been treated with intensive chemotherapy, and for whom cytogenetic and molecular genetic data on the mutational status of *FLT3*, *NPM1* and all nine MRG mutations were available. Data sets were compiled from 9 data providers across Europe. Some of the patients were part of previously published clinical trials or retrospective analyses [23–27]. Only 16 patients received FLT3 inhibitors during induction. Patient data uploaded to the HARMONY Platform underwent a rigorous double brokerage pseudonymization process adhering to the General Data Protection Regulation (GDPR). Subsequently, the data were harmonized and converted using the Observational Medical Outcomes Partnership (OMOP) Common Data Model.

The study was performed in accordance with the Declaration of Helsinki and received approval from the former HARMONY Alliance steering committee and AML working group. The former HARMONY Alliance research project underwent review and approval by the Medicinal Research Ethics Committee of the University of Salamanca (PI 2018 10 128). The HARMONY Alliance Foundation has established an ethical and data-protection framework for the secondary use of data, including *de facto* anonymization. Prior written informed consent for data use had been obtained from all patients at respective HARMONY Alliance partner institutions.

### Genetic analysis

Cytogenetic data were collected at each respective center following local guidelines and annotated according to the ISCN-2020 criteria [28]. All included datasets were also analyzed by the data providers using various molecular genetic panels that cover commonly mutated genes in AML. Variants were included as reported by the data providers. VAFs were harmonized as decimals and adjusted for sex, with values divided by 2 for MRG mutations located on the X chromosome in male subjects (*BCOR, STAG2, ZRSR2)*.

### Statistical analysis

Complete remission (CR), complete remission with incomplete blood count recovery (CRi) and relapse were (re-) defined according to ELN 2022 criteria [4]. OS was calculated from diagnosis to death or last follow-up. Relapse-free survival (RFS) was calculated as the time from CR/CRi to death, relapse, or last follow-up, whichever occurred first. Patients for whom the date of response was missing were excluded for RFS analysis but included in the analysis of OS. Follow-up was censored at 10 years. The median follow-up for survival was calculated using the reverse Kaplan-Meier estimate [29].

All statistical analyses were performed using R (packages cowplot, dplyr, ggplot2, ggsurvfit, gtsummary, mice, survival, survivalAnalysis, survminer, tidyverse).

Categorical variables were compared between two independent groups using the χ^2^ test and continuous variables were compared using the Wilcoxon rank sum test.

RFS and OS distributions were estimated using the Kaplan-Meier method. Uni- and multivariable analyses were performed using a Cox proportional hazards model. For multivariable analyses multiple data imputation was performed using either logistic regression for binary variables or predictive mean matching for continuous variables, employing chained equations using five imputations. For multivariable analysis we considered the known risk factors at baseline age, WBC, karyotype, *TP53* and *NPM1* mutational status.

## Results

### Mutational landscape of *FLT3*-ITD positive AML

This study included 4 078 intensively treated AML patients from the HARMONY Platform (Supplementary Fig. S1). The median follow-up was 6.2 years. 862 patients (21%) were *FLT3*-ITD positive (pos) at the time of diagnosis. Patient characteristics reflected known characteristics of *FLT3*-ITD positive patients with a higher WBC count and a higher prevalence of female patients (Supplementary Table S1) **[**16]. Among *FLT3*-ITD^pos^ AML, 171 patients (20%) carried at least one MRG mutation, among which 108 (63%) had only one, 44 (26%) had two and 19 (11%) had ≥ 3 MRG mutations. 491 patients (57%) were *NPM1* mutated (mut).

The most common MRG mutations were found in *RUNX1* (77, 45% of all patients with MRG mutations), *SRSF2* (37, 22%), *STAG2* (32, 19%) and *ASXL1* (29, 17%). The median VAF ranged from 0.4 for *ASXL1* to 0.6 for *ZRSR2* (Supplementary Table S2 and S3).

Supplementary Figure S2 and Table S2 illustrate the distribution of co-mutations within the *FLT3*^pos^ cohort. MRG mutations occurred in 125 (34%) of *FLT3*-ITD/*NPM1* wildtype (wt) patients, whereas *FLT3*-ITD^pos^/*NPM1*^mut^ patients were less likely to carry MRG mutations (46 patients, 9%, *p* < 0.001, Supplementary Table S4). *DNMT3A* mutations were associated with *NPM1* mutations and thus were more common in patients without MRG mutation (*p* < 0.001). Comparing the mutational landscape between *FLT3*-ITD^pos^/*NPM1*^mut^ and *FLT3*-ITD^pos^/*NPM1*^wt^ patients, *NRAS* mutations as well as *ASXL1, BCOR, EZH2, RUNX1* and *U2AF1* were more common in the *FLT3*-ITD^pos^/*NPM1*^wt^ group (Supplementary Table S5).

### Clinical characteristics and prognostic impact of MRG mutations

Considering the *FLT3*-ITD^pos^ AML cohort, patients with MRG co-mutation were older (median age 57 vs. 51 years), more likely to be male (61% vs. 46%), and had a lower WBC at diagnosis (26.6 vs. 43.9 ×10^9^/L) compared to MRG^wt^ patients, consistent with previously described characteristics of MRG^mut^ patients [7, 9, 30]. The likelihood to achieve CR/CRi after two cycles of chemotherapy was lower in MRG^*mut*^ patients (74% vs. 82%) (Table 1).

**Table 1:** Patient characteristics of all AML patients with *FLT3*-ITD.

Characteristic	All (*n* = 862)	MRG mutation (*n* = 171)	No MRG mutation (*n* = 691)	*p*
Age at diagnosis (years)				<0.001
Median	51	57	49.9	
Range	18 – 75	20 – 75	18 – 75	
Patient sex				<0.001
Male – no. (%)	394 (46)	104 (61)	290 (42)	
Female – no. (%)	468 (64)	67 (49)	401 (58)	
ECOG PS at diagnosis				0.45
0–1 – no. (%)	364 (42)	75 (44)	289 (42)	
>1 – no. (%)	93 (11)	22 (13)	71 (10)	
No information –no. (%)	405 (47)	74 (43)	331 (48)	
WBC at diagnosis x10^9^/L				0.001
Median	43.9	26.64	47.79	
Range	0.2 – 549.5	0.7 – 406	0.2 – 549.5	
No information –no. (%)	43 (5)	9 (5)	34 (5)	
Hgb at diagnosis g/dl				0.32
Median	9	8.85	9	
Range	2.5 – 16	2.7 – 14.2	2.5 – 16	
No information –no. (%)	52 (6)	9 (5)	43 (6)	
Platelets at diagnosis x10^9^/L				0.092
Median	57	48	58	
Range	2–916	2–407	3–916	
No information –no. (%)	137 (16)	30 (18)	107 (15)	
Favorable risk cytogenetics – no (%)	43 (5)	8 (5)	35 (5)	0.99
Intermediate risk cytogenetics – no (%)	772 (90)	153 (90)	619 (90)	1
Adverse risk cytogenetics – no (%)	47 (5)	10 (6)	37 (5)	0.95
CR/CRi – no. (%)	691 (80)	127 (74)	564 (82)	0.04

*CR* complete remission, *CRi* complete remission with incomplete hematological recovery, *Hgb* hemoglobin, *ECOG PS* Eastern Cooperative Oncology Group Performance Status, *WBC* white blood cell count.

**Fig. 1:**
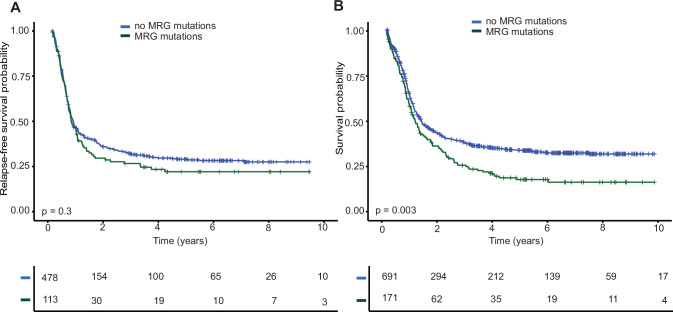
Outcome of *FLT3*-ITD ^pos^ patients stratified by MRG mutations. Relapse-Free Survival (RFS) **A** and Overall Survival (OS) **B** of *FLT3*-ITD positive patients with and without myelodysplasia-related gene (MRG) co-mutations.

We first compared RFS and OS of *FLT3*-ITD^pos^ patients stratified by the presence of MRG mutations in univariate and multivariable analyses. While no difference in RFS was observed (HR 1.15, 95%CI 0.90–1.46, *p* = 0.3, Fig. 1A), OS was significantly shorter for MRG^mut^ patients (HR 1.36, 95%CI 1.11–1.66, *p* = 0.003, Fig. 1B) in univariate analysis. Importantly, in multivariable analysis for OS, only high WBC counts, age and *NPM1* co-mutations were independently associated with OS, whereas the presence of MRG mutations was not (HR 1.14, 95%CI 0.92–1.42, *p* = 0.2) (Table 2). Of note, although *TP53* mutations did not emerge as an independent adverse factor in multivariable analysis, the number of patients with *TP53* mutations was small (*n* = 10) in this cohort, thus precluding definite conclusions.

**Table 2:** Uni- and multivariable analyses for overall survival in the total cohort of *FLT3*-ITD positive patients (*n* = 862).

	Univariate				Multivariable			
Variable	HR^#^	95% CI LL	95% CI UL	*p*	HR^#^	95%CI LL	95% CI UL	*p*
MRG mutant vs. wildtype	1.36	1.11	1.66	0.003	1.14	0.92	1.42	0.2
WBC at diagnosis (increase of 1, log_2_, x10^9^/L)	1.12	1.07	1.18	<0.001	1.13	1.08	1.18	<0.001
Age (increase by 10)	1.22	1.14	1.30	<0.001	1.22	1.14	1.30	<0.001
*TP53* mutant vs. wildtype	2.03	1.01	4.08	0.047	1.54	0.76	3.12	0.2
*NPM1* mutant vs. wildtype	0.75	0.63	0.89	<0.001	0.66	0.55	0.80	<0.001
Favorable risk cytogenetics yes vs. no	0.69	0.44	1.06	0.09	0.66	0.42	1.04	0.075
Adverse risk cytogenetics yes vs. no	1.25	0.87	1.81	0.2	-	-	-	-

^#^Hazard ratios greater than or less than 1 indicate an increased or decreased risk, respectively, of an event for the first category listed.

*CI* confidence interval, *HR* hazard ratio, *LL* lower limit, *MRG* myelodysplasia-related gene, *UL* upper limit, *WBC* white blood cell count.

Thus, MRG co-mutations in *FLT3*-ITD^pos^ AML do not alter the clinical outcome, and do not confer independent adverse prognosis in the overall *FLT3*-ITD^pos^ AML cohort.

In an exploratory, univariate analysis we considered *FLT3*-ITD^pos^ MRG^mut^ patients in the context of the ELN 2022 risk classification. These patients displayed RFS and OS outcomes comparable to those classified as ELN adverse risk patients (RFS, HR 1.02, 95%CI 0.91–1.14, *p* = 0.8; OS, HR 0.98, 95%CI 0.89–1.08, *p* = 0.7) and significantly worse than both ELN favorable and intermediate risk groups (ELN favorable risk: RFS, HR 1.23, 95%CI 1.16–1.3, *p* < 0.001; OS, HR 1.34, 95%CI 1.27–1.41, *p* < 0.001; ELN intermediate risk: RFS, HR 1.18, 95%CI 1.09–1.28, *p* < 0.001; OS, HR 1.24, 95%CI 1.16–1.33, *p* < 0.001, Supplementary Fig. S3).

### Prognostic impact of MRG mutations by *NPM1* mutational status

Since MRG mutations were overrepresented in the *FLT3*-ITD^pos^/*NPM*1^wt^ cohort, and *NPM1* mutations were one of the strongest favorable prognostic markers for OS in multivariable analysis, we next evaluated the prognostic impact of MRG mutations stratified by *NPM1* mutations.

The VAF of MRG mutations did not differ between *NPM1*^wt^and *NPM1*^mut^patients **(**Supplementary Table S3**)**. In the *NPM1*^wt^ subgroup, patients with MRG mutations were older, more often male, and had lower platelet counts at diagnosis **(**Supplementary Table S6**)**.

**Fig. 2:**
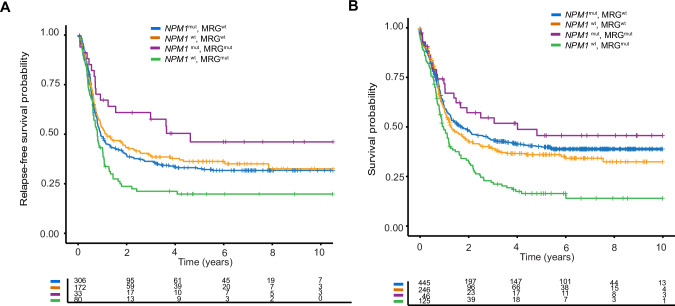
Outcome of *FLT3*-ITD^pos^ patients stratified by *NPM1* mutations. Relapse-Free Survival (RFS) **A** and Overall Survival (OS) **B** of all *FLT3*-ITD positive patients stratified by *NPM1* and myelodysplasia-related gene (MRG) mutations.

MRG mutations were present in 125 (34%) of all FLT3-ITD^pos^/NPM1^wt^ patients. Both RFS and OS were significantly shorter in *FLT3*-ITD^pos^/*NPM1*^*wt*^ patients with MRG mutations compared to patients without MRG mutations in univariate analysis (RFS, HR: 1.58, 95%CI 1.17–2.13, *p* = 0.003; OS, HR: 1.55, 95%CI 1.20–1.99, *p* < 0.001, Fig. 2A, B). Strikingly, MRG mutations were shown to have an independent prognostic role on RFS and OS in multivariable analysis (RFS: HR 1.37, 95%CI 1.01 – 1.88, *p* = 0.046, OS: HR 1.34, 95%CI 1.02–1.74, *p* = 0.032), alongside other established prognostic factors in AML such as elevated WBC count, older age, and favorable risk cytogenetics (Table 3).

**Table 3:** Multivariable analysis of relapse-free survival and overall survival in *FLT3*-ITD^pos^/*NPM1*^wt^ patients (*n* = 371).

	Univariate				Multivariable			
RFS	HR^#^	95% CI LL	95% CI UL	*p*	HR^#^	95% CI LL	95% CI UL	*p*
MRG mutant vs. wildtype	1.58	1.17	2.13	0.003	1.37	1.01	1.88	0.046
WBC at diagnosis (increase of 1, log_2_, x10^9^/L)	1.13	1.05	1.21	0.002	1.12	1.04	1.21	0.002
Age (increase by 10)	1.17	1.05	1.3	0.004	1.14	1.02	1.27	0.019
*TP53* mutant vs. wildtype	0.56	0.08	4.01	0.6	-	-	-	-
Favorable risk cytogenetics yes vs. no	0.38	0.22	0.63	<0.001	0.43	0.26	0.73	0.002
Adverse risk cytogenetics yes vs. no	1.07	0.65	1.76	0.8	-	-	-	-

OS								
MRG mutant vs. wildtype	1.55	1.20	1.99	<0.001	1.34	1.02	1.74	0.032
WBC at diagnosis (increase of 1, log_2_, x10^9^/L)	1.11	1.04	1.18	0.002	1.12	1.05	1.19	<0.001
Age (increase by 10)	1.28	1.17	1.4	<0.001	1.25	1.14	1.37	<0.001
*TP53* mutant vs. wildtype	1.02	0.25	4.11	1	-	-	-	-
Favorable risk cytogenetics yes vs. no	0.51	0.32	0.81	0.004	0.62	0.39	0.99	0.047
Adverse risk cytogenetics yes vs. no	1.02	0.69	1.50	1	-	-	-	-

^#^Hazard ratios greater than or less than 1 indicate an increased or decreased risk, respectively, of an event for the first category listed.

*CI* confidence interval, *HR* hazard ratio, *LL* lower limit, *MRG* myelodysplasia-related gene, *OS* overall survival, *RFS* relapse-free survival, *UL* upper limit, *WBC* white blood cell count.

In the context of the ELN 2022 risk classification, RFS of *FLT3*-ITD^pos^/*NPM1*^*wt*^ patients with MRG mutation was worse than that of the ELN 2022 adverse risk group in univariate analysis (HR 1.18, 95% CI 1.04–1.34, *p* = 0.01) (Supplementary Fig. S4A). OS of *NPM1*^wt^ patients with MRG mutation was comparable to that of the ELN 2022 adverse risk group (HR 1.09, 95% CI 0.98–1.21, *p* = 0.1), while long-term OS of *NPM1*^wt^ patients without MRG mutation was significantly better than that of the ELN adverse risk group (HR 0.76, 95% CI 0.64–0.91, *p* = 0.002, Supplementary Fig. S4B).

In the *NPM1*^mut^ subgroup, AML with MRG mutations was rare (9% of all FLT3-ITD^pos^/NPM1^mut^ patients) and was associated with older age, with no difference for sex and blood counts versus patients without MRG mutations (Supplementary Table S7).

In *FLT3*-ITD^pos^/*NPM1*^mut^patients MRG mutations were not an independent factor for RFS and OS in multivariable analysis (Supplementary Table S8).

In the context of the ELN 2022 risk classification, RFS of *NPM1*^mut^/MRG^mut^ patients was comparable to that of the ELN 2022 favorable risk group (HR 1.03, 95% CI 0.91–1.16, *p* = 0.7) in univariate analysis, RFS of *NPM1*^mut^/ MRG^wt^ patients was comparable to that of the ELN adverse risk group (HR 0.92, 95% CI 0.78–1.08, *p* = 0.3, Supplementary Fig. S4C). OS of *FLT3*-ITD^pos^/*NPM1*^mut^/MRG^mut^ patients was comparable to that of the ELN 2022 intermediate risk group (HR 1.01, 95% CI 0.87–1.16, *p* = 1), while OS of *NPM1*^mut^*/* MRG^wt^ patients was significantly worse than that of the ELN 2022 intermediate risk group (HR 1.15, 95% CI 1.05–1.24, *p* = 0.001), but significantly better than that of the ELN 2022 adverse risk group (HR 0.67, 95% CI 0.58–0.77, *p* < 0.001, Supplementary Fig. S4D).

Thus, MRG mutations are an independent adverse risk factor for RFS and OS in *FLT3*-ITD^pos^/*NPM1*^*wt*^ patients, but not in *FLT3*-ITD^pos^/*NPM1*^mut^ patients.

*FLT3*-ITD^pos^/*NPM1*^wt^ patients with and without MRG mutations both showed significant improvement of OS when undergoing alloHCT in first CR/CRi (p = 0.036 and p = 0.002, respectively, Supplementary Fig. S5).

### Impact of *FLT3*-ITD allelic ratio

We also evaluated the prognostic impact of the AR of *FLT3*-ITD mutations, classifying patients with an AR ≥ 0.5 as high AR (*n* = 525, 61%) and those with an AR < 0.5 as low AR (*n* = 231, 27%). For 106 patients (12%) no allelic ratio was available.

**Fig. 3:**
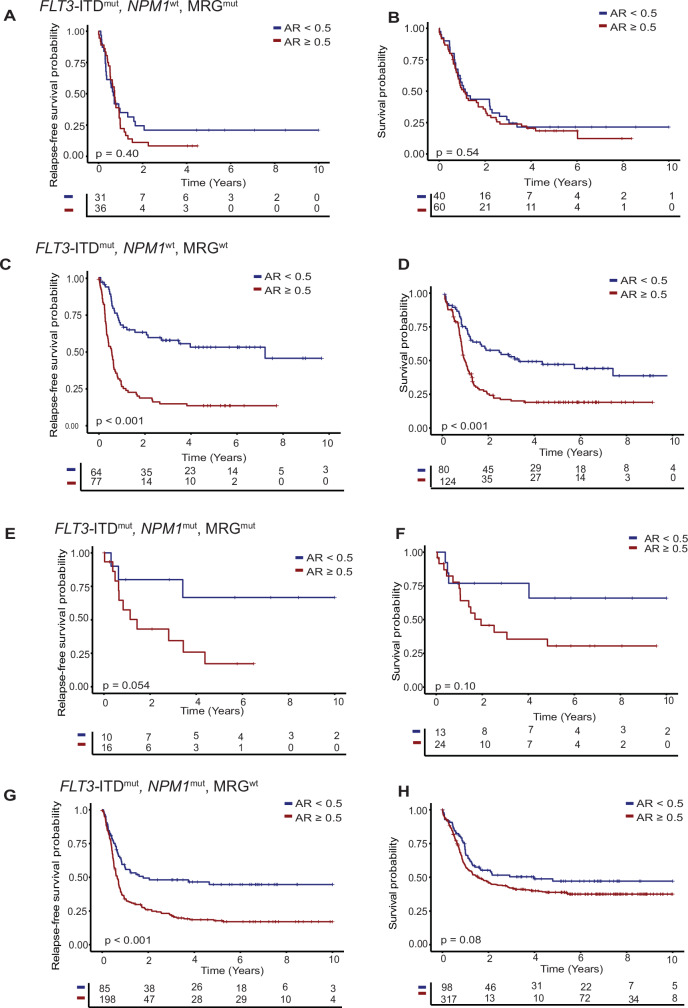
Impact of *FLT3*-ITD allelic ratio on outcome. RFS (left) and OS (right) of *NPM1*^wt^/MRG^mut^ **A, B**, *NPM1*^wt^/ MRG^wt^ **C, D**, *NPM1*^mut^/ MRG^mut^ **E, F** and *NPM1*^mut^/ MRG^wt^ patients stratified by *FLT3*-ITD allelic ratio **G, H** (AR).

In the *NPM1*^wt^ cohort, a high *FLT3*-ITD AR did not further stratify the outcome of MRG mutant patients in uni- and multivariable analyses (Fig. 3A, B, Supplementary Table S9), but was associated with a shorter RFS and OS in MRG^wt^ patients (RFS: HR 2.92, 95%CI 1.95–4.37, *p* < 0.001; OS: 1.75, 95%CI 1.24–2.48, *p* = 0.002, Fig. 3C, D, Supplementary Table S9).

In the *NPM1*^mut^ cohort, a high *FLT3*-ITD AR was associated with shorter RFS and OS in MRG^mut^ patients (RFS: HR 3.55, 95%CI 1.14–11.1, *p* = 0.029; OS: HR 3.58, 95%CI 1.20–10.7, *p* = 0.022, Fig. 3E, F, Supplementary Table S9), and a shorter RFS in MRG^wt^ patients (RFS: HR 1.91, 95%CI 1.37–2.68, *p* < 0.001; OS: HR 1.12, 95%CI 0.81–1.54, *p* = 0.5, Fig. 3G, H, Supplementary Table S9). Thus, the *FLT3*-ITD AR had no impact in the patient group with the worst prognosis, i.e. *FLT3*-ITD^pos^/*NPM1*^wt^ patients with MRG mutation, while it seems to play a prognostic role in the other patient subgroups.

### Prognostic impact of number of MRG mutations and individual MRG mutations in *FLT3*-ITD^pos^/*NPM1*^wt^ AML

Given prior reports that the number of MRG mutations may influence prognosis [9], we assessed the impact of having one versus multiple MRG mutations on RFS and OS. In the *FLT3-ITD*^pos^/*NPM1*^mut^ subgroup, no differences in RFS or OS were observed between patients with one and those with two MRG mutations (Supplementary Figs. S6A and S6B). In the *FLT3-ITD*^pos^/*NPM1*^wt^ subgroup, patients with a single MRG mutation had RFS comparable to MRG-negative patients, whereas those with ≥2 MRG mutations had significantly worse RFS. A similar pattern was seen for OS, where patients with one MRG mutation showed a trend toward inferior survival compared with MRG-negative patients, while those with ≥2 mutations had significantly worse OS (Supplementary Figs. S6C and S6D).

Recent studies have suggested that individual MRG mutations differ in prognostic significance, with only *ASXL1, RUNX1, SF3B1*, and *U2AF1* being associated with adverse outcomes [31]. Therefore, we analysed the effect of each mutation separately on RFS and OS in *FLT3*-ITD^pos^/*NPM1*^wt^ patients. Although the number of patients carrying each individual mutation was small, our analysis suggests that in *FLT3*-ITD^pos^/*NPM1*^wt^ patients, all MRG mutations contribute to the adverse prognostic effect on RFS except *ASXL1* and *STAG2*, while all MRG mutations contribute to the adverse prognostic effect on OS except *BCOR* and *EZH2* (Supplementary Fig. S7).

## Discussion

In this HARMONY Alliance cohort of 862 *FLT3*-ITD^pos^ AML patients, 171 (20%) harbored at least one MRG mutation. These mutations were associated with known characteristics including older age, male sex, lower WBC count, and *NPM1* wildtype status. MRG mutations had no independent prognostic impact on RFS and OS in the overall cohort of *FLT3*-ITD AML patients. However, among *FLT3-*ITD^pos^/*NPM1*^wt^ patients, MRG mutations occurred in 125 (34%) patients and were independently associated with inferior RFS and OS. In contrast, MRG mutations were found in only 26 (9%) of *FLT3-ITD*^*pos*^*/NPM1*^mut^ patients and did not confer adverse prognostic significance.

According to the 2022 ELN recommendation, patients with *FLT3*-ITD and an MRG co-mutation are classified as ELN 2022 adverse risk [4]. *FLT3*-ITD^pos^/*NPM1*^wt^ patients with or without MRG mutations overall had a similar prognosis to ELN 2022 adverse risk patients. Importantly, patients with MRG mutations had worse long-term RFS and OS compared to patients without MRG mutation. Thus, our data confirm the classification of ELN 2022 in *FLT3*-ITD^pos^ /*NPM1*^wt^patients, in which MRG co-mutations assign an adverse risk.

In contrast, patients *with FLT3*-ITD^pos^/*NPM1*^mut^/MRG^mut^ AML had a RFS similar to those with favorable risk AML, and an OS similar to those with ELN intermediate risk AML. These data suggest that the presence of MRG mutations in *FLT3*-ITD^pos^/*NPM1*^mut^ AML do not justify assignment to the ELN adverse risk category. Three-year OS of *FLT3*-ITD^pos^/*NPM1*^mut^/MRG^mut^ patients was 55%, while it was 22% for *FLT3*-ITD^pos^/*NPM1*^wt^/MRG^mut^ patients. Although a 33% 3-year OS difference is clinically relevant, the outcome is still suboptimal. This may reflect historical treatment patterns, including the absence of FLT3 inhibitors and lower transplantation rates (27% in our overall cohort) compared with current practice. The missing impact of MRG mutations in the context of co-occurring *NPM1* mutations aligns with reports that MRG mutations do not confer an adverse outcome in *NPM1* mutated AML [12, 14, 32, 33]. Thus, our findings suggest that *NPM1* co-mutations may mitigate the adverse effect of MRG mutations.

Our results suggest that allelic ratio may be helpful to stratify patients with *NPM1* co-mutations but fails to show additional value in the subgroup with the worst outcome, *FLT3*-ITD^pos^/MRG^mut^/*NPM1*^wt^ patients. Our subgroup analyses suggest that two or more MRG mutations confer an even worse prognosis in *FLT3*-ITD^pos^/*NPM1*^wt^ patients and that a broad spectrum of individual MRG mutations contribute to the adverse prognostic effect.

Our study has several limitations. The prognosis of *FLT3* mutant patients has significantly improved with the use of FLT3i [19–21]. However, we were unable to evaluate the impact of these agents, as only a small subset of patients in our cohort received FLT3i therapy. Because FLT3i have become the standard of care for patients with *FLT3*-ITD mutations, the immediate translatability of our cohort may be limited. However, a post-hoc analysis of the QUANTUM-First trial suggests that patients with MRG mutations derive only limited benefit from the addition of quizartinib. In this analysis, outcomes with quizartinib versus placebo were compared among *FLT3*-ITD–mutated patients carrying at least one mutation in one of nine MR-associated genes. Overall survival was similar between the treatment arms (HR 0.998; 95% CI, 0.72–1.39), indicating that our findings may still be applicable within the current FLT3-inhibitor treatment landscape [20, 34].

As a retrospective cohort study, it does not control for selection bias. Moreover, MRD evaluation serves as a validated prognostic indicator in *FLT3*-mutated AML, with MRD negativity following two cycles of chemotherapy correlating with superior clinical outcomes [35]. As MRD monitoring was not routinely performed in our cohort, we could not analyze its prognostic significance. Lastly, we focus on intensively-treated patients, whereas the prognostic impact of these mutations in non-intensively treated patients could be different [36].

In summary, we present a detailed analysis of the prognostic impact of MRG mutations in patients with *FLT3*-ITD^pos^ AML suggesting a nuanced role of MRG mutations in *FLT3*-ITD^pos^ AML. Our findings support a refinement of the current risk stratification: While MRG mutations are associated with poor outcomes in the absence of *NPM1* co-mutations, aligning with the ELN 2022 classification, they lack prognostic impact when co-occurring with *NPM1* mutations.

## Supplementary information

Supplementary Information to manuscript

## Data Availability

Data is available within the HARMONY Alliance Foundation and can be accessed upon reasonable request to the HARMONY Alliance Foundation Board.
