# Supplementary material for: Prognostic impact of myelodysplasia-related gene mutations in FLT3-ITD-mutated acute myeloid leukemia
Source: Leukemia. 2026 Feb 9;40(3):622–9. doi: 10.1038/s41375-026-02874-w (PMC12960240; doi:10.1038/s41375-026-02874-w)
Supplement: Supplementary file 1 — Supplementary Information to manuscript [file 41375_2026_2874_MOESM1_ESM.docx]

**Prognostic impact of myelodysplasia-related gene mutations in acute myeloid leukemia with *FLT3*-ITD**

Rabea Mecklenbrauck, Angela Villaverde Ramiro, Eric Sträng, Razif Gabdoulline, Javier Martinez Elicegui, Marta Sobas,  Lisa Pleyer, Amin Turki, Maria Teresa Voso, Axel Benner, Alberto Hernández-Sánchez, Jesse M Tettero, Laura Tur Gimenez, Klaus H. Metzeler, Guadalupe Oñate, Sören Lehmann, Brian JP Huntly, Ian Thomas, Felicitas R Thol, Florian H Heidel, Peter JM Valk, Konstanze Döhner, Torsten Haferlach, Kenneth I Mills, Hartmut Döhner, Gastone Castellani, Gert J Ossenkoppele, Jesus María Hernández-Rivas, Lars Bullinger, Michael Heuser

**Supplementary Information**

**Supplementary Table S1**: Patient characteristics of all AML included patients and stratified by *FLT3*-ITD mutation status.

| **Characteristic** | **All (n=4078)** | ***FLT3*-ITD positive (n=862)** | ***FLT3*-ITD negative (n=3216)** | **p** |
| --- | --- | --- | --- | --- |
| Age at diagnosis (years) |  |  |  | 0.34 |
| Median | 52 | 51 | 52 |  |
| Range | 18 – 75 | 18 – 75 | 18 – 75 |  |
| Patient sex |  |  |  | <0.001 |
| Male – no. (%) | 2146 (54) | 394 (46) | 1752 (54) |  |
| Female – no. (%) | 1932 (47) | 468 (54) | 1464 (46) |  |
| ECOG PS at diagnosis |  |  |  | <0.001 |
| 0–1 – no. (%) | 1637 (40) | 364 (42) | 1273 (40) |  |
| >1 – no. (%) | 324 (8) | 93 (11) | 231 (7) |  |
| No information –   no. (%) | 2117 (52) | 405 (47) | 1712 (53) |  |
| WBC at diagnosis x10^9^/L |  |  |  | <0.001 |
| Median | 16 | 43.9 | 11.95 |  |
| Range | 0.1 – 550 | 0.2 – 550 | 0.1 – 533 |  |
| No information –   no. (%) | 256 (6) | 43 (5) | 213 (6) |  |
| Hgb at diagnosis g/dl |  |  |  | 0.52 |
| Median | 9 | 9 | 9 |  |
| Range | 2.5 – 17.6 | 2.5 – 16 | 1.5 – 17.6 |  |
| No information –   no. (%) | 321 (8) | 52 (6) | 269 (8) |  |
| Platelets at diagnosis x10^9^/L |  |  |  | 0.83 |
| Median | 56 | 57 | 56 |  |
| Range | 1 – 2013 | 2 – 916 | 1 – 2013 |  |
| No information –   no. (%) | 767 (19) | 137 (16) | 630 (20) |  |
| CR/CRi – no. (%) | 3218 (79) | 689 (80) | 2526 (79) | 0.33 |

Abbreviations: CR, complete remission; CRi, complete remission with incomplete hematological recovery; Hgb, hemoglobin; ECOG PS, Eastern Cooperative Oncology Group Performance Status; WBC, white blood cell count

**Supplementary Table S2**: Incidence and variant allele frequency (VAF) of MRG mutations in *FLT3*-ITD^pos^ AML.

| **Mutation** | **Mutation frequency and VAF** |
| --- | --- |
| *ASXL1*, n (%)  Median VAF (range) | 29 (17)  0.3986 (0.0407 – 0.53) |
| *BCOR*, n (%)  Median VAF (range) | 27 (16)  0.426 (0.05 – 0.9357) |
| *EZH2*, n (%)  Median VAF (range) | 15 (9)  0.48 (0.1102 – 0.95) |
| *RUNX1*, n (%)  Median VAF (range) | 77 (45)  0.434 (0.0453 – 0.9586) |
| *SF3B1*, n (%)  Median VAF (range) | 13 (8)  0.41 (0.0265 – 0.6126) |
| *SRSF2*, n (%)  Median VAF (range) | 37 (22)  0.42 (0.01 – 0.76) |
| *STAG2*, n (%)  Median VAF (range) | 32 (19)  0.4873 (0.0701 – 1) |
| *U2AF1*, n (%)  Median VAF (range) | 20 (12)  0.41 (0.03 – 0.75) |
| *ZRSR2*, n (%)  Median VAF (range) | 8 (5)  0.5982 (0.0686 – 0.9615) |

**Supplementary Table S3:** Variant allele frequency of MRG mutations and *NPM1.*

|  | **All (n=862)** | ***NPM1* mut (n=491)** | ***NPM1* wt (n=371)** | **p** |
| --- | --- | --- | --- | --- |
| *NPM1* VAF, median (range) | 0.37 (0.047 – 0.69) | 0.37 (0.047 – 0.69) | na | na |
| *ASXL1* VAF, median (range) | 0.4 (0.041 – 0.53) | 0.29 (0.041 – 0.5) | 0.40 (0.08 – 0.53) | 0.49 |
| *BCOR* VAF, median (range) | 0.43 (0.05 – 0.94) | 0.43 (0.4 – 0.53) | 0.427 (0.05 – 0.94) | 0.97 |
| *EZH2* VAF, median (range) | 0.48 (0.11 – 0.95) | 0.39 (na) | 0.49 (0.11 – 0.95) | 0.4 |
| *RUNX1* VAF, median (range) | 0.43 (0.045 – 0.96) | 0.44 (0.43 – 0.46) | 0.43 (0.045 – 0.96) | 0.81 |
| *SF3B1* VAF, median (range) | 0.41 (0.027 - 0.61) | 0.4 (0.027 - 0.49) | 0.43 (0.04 - 0.61) | 0.44 |
| *SRSF2* VAF, median (range) | 0.42 (0.01 - 0.76) | 0.39 (0.01 - 0.76) | 0.44 (0.22 - 0.71) | 0.17 |
| *STAG2* VAF, median (range) | 0.49 (0.07 - 1) | 0.46 (0.07 - 0.95) | 0.77 (0.08 - 1) | 0.11 |
| *U2AF1* VAF, median (range) | 0.41 (0.03 - 0.75) | 0.16 (0.05 - 0.27) | 0.42 (0.03 - 0.75) | 0.097 |
| *ZRSR2* VAF, median (range) | 0.6 (0.069 - 0.96) | 0.6 (0.504- 0.69) | 0.56 (0.069 - 0.96) | 1 |

Abbreviations: na, not applicable; VAF, variant allele frequency

**Supplementary Table S4**: Co-mutations in *FLT3*-ITD^pos^ AML by MRG mutation status.

| **Co-mutation** | ***FLT3*-ITD with MRG mutation (n=171)** | ***FLT3*-ITD no MRG mutation (n=691)** | ***p **** |
| --- | --- | --- | --- |
| *DNMT3A*, n (%) | 44 (26) | 309 (45) | <0.001 |
| *FLT3-TKD*, n (%) | 16 (9) | 55 (8) | 0.73 |
| *GATA2*, n (%) | 3 (2) | 21 (3) | 0.73 |
| *IDH1*, n (%) | 6 (4) | 54 (8) | 0.33 |
| *IDH2*, n (%) | 20 (12) | 70 (10) | 0.73 |
| *KIT*, n (%) | 5 (3) | 16 (2) | 0.85 |
| *KRAS*, n (%) | 5 (3) | 14 (2) | 0.73 |
| *MYC*, n (%) | 2 (1) | 18 (3) | 0.73 |
| *NPM1*, n (%) | 46 (30) | 445 (64) | <0.001 |
| *NRAS*, n (%) | 20 (12) | 50 (7) | 0.33 |
| *PHF6*, n (%) | 6 (4) | 11 (2) | 0.40 |
| *PTPN11*, n (%) | 14 (8) | 32 (5) | 0.33 |
| *RAD21*, n (%) | 10 (6) | 49 (7) | 0.73 |
| *SMC1A*, n (%) | 1 (1) | 15 (2) | 0.40 |
| *SMC3*, n (%) | 2 (1) | 15 (2) | 0.73 |
| *TET2*, n (%) | 31 (18) | 95 (14) | 0.40 |
| *WT1*, n (%) | 18 (11) | 107 (15) | 0.36 |

*p-value corrected with Benjamini-Hochberg method for multiple testing

**Supplementary Table S5**: Co-mutations in *FLT3*-ITD^pos^/*NPM1*^wt^ and *FLT3*-ITD^pos^/*NPM1*^wt^ patients.

| **Co-mutation** | ***FLT3*-ITD^pos^/*NPM1*^wt^ (n=371)** | ***FLT3*-ITD^pos^/*NPM1*^mut^ (n=491)** | ***p **** |
| --- | --- | --- | --- |
| *DNMT3A*, n (%) | 81 (22) | 271(55) | <0.001 |
| *FLT3-TKD*, n (%) | 34 (9) | 37 (8) | 0.52 |
| *GATA2*, n (%) | 7 (2) | 17 (3) | 0.3 |
| *IDH1*, n (%) | 21 (6) | 39 (8) | 0.3 |
| *IDH2*, n (%) | 27 (7) | 63 (13) | 0.02 |
| *KIT*, n (%) | 16 (4) | 5 (1) | 0.008 |
| *KRAS*, n (%) | 13 (4) | 6 (1) | 0.062 |
| *MYC*, n (%) | 8 (2) | 12 (2) | 0.98 |
| *NRAS*, n (%) | 44 (12) | 26 (5) | 0.002 |
| *PHF6*, n (%) | 12 (3) | 5 (1) | 0.064 |
| *PTPN11*, n (%) | 12 (3) | 34 (7) | 0.05 |
| *RAD21*, n (%) | 10 (3) | 49 (10) | 0.002 |
| *SMC1A*, n (%) | 7 (2) | 9 (2) | 1 |
| *SMC3*, n (%) | 2 (1) | 15 (3) | 0.04 |
| *TET2*, n (%) | 39 (11) | 87 (17) | 0.009 |
| *WT1*, n (%) | 69 (19) | 56 (11) | 0.009 |
| *ASXL1*, n (%) | 25 (7) | 4 (1) | <0.001 |
| *BCOR*, n (%) | 24 (6) | 3 (1) | <0.001 |
| *EZH2*, n (%) | 14 (4) | 1 (0.2) | <0.001 |
| *RUNX1,* n (%) | 74 (20) | 3 (1) | <0.001 |
| *SF3B1,* n (%) | 8 (2) | 8 (2) | 0.33 |
| *SRSF2, n (%)* | 21 (6) | 16 (3) | 0.18 |
| *STAG2, n (%)* | 17 (5) | 15 (3) | 0.36 |
| *U2AF1,* n (%) | 17 (5) | 3 (1) | 0.001 |
| *ZRSR2,* n (%) | 6 (2) | 2 (0.4) | 0.2 |

*p-value corrected with Benjamini-Hochberg method for multiple testing

**Supplementary Table S6**: Characteristics of *FLT3*-ITD^mut^/*NPM1*^wt^ patients by MRG mutation status.

| **Characteristic** | **All (n=371)** | **MRG mutation (n=125)** | **No MRG mutation (n=246)** | **p** |
| --- | --- | --- | --- | --- |
| Age at diagnosis (years) |  |  |  | <0.001 |
| Median | 49 | 59 | 45 |  |
| Range | 18 – 75 | 20 – 75 | 18 – 75 |  |
| Patient sex |  |  |  | 0.005 |
| Male – no. (%) | 210 (57) | 84 (67) | 126 (51) |  |
| Female – no. (%) | 161 (43) | 41 (33) | 120 (49) |  |
| ECOG PS at diagnosis |  |  |  | 0.90 |
| ≤ 1 – no. (%) | 172 (46) | 59 (46) | 113 (46) |  |
| > 1 – no. (%) | 42 (11) | 15 (12) | 27 (11) |  |
| No information, no. (%) | 157 (42) | 51 (41) | 106 (44) |  |
| WBC at diagnosis x10^9^/L |  |  |  | 0.07 |
| Median | 41.4 | 25.1 | 52.45 |  |
| Range | 0.7 – 549.5 | 0.9 – 302 | 0.7 – 549.5 |  |
| No information, no. (%) | 18 (5) | 4 (3) | 14 (6) |  |
| Hgb at diagnosis g/dl |  |  |  | 0.1 |
| Median | 9 | 8.8 | 9.05 |  |
| Range | 2.5 – 14.6 | 4.2 – 13.9 | 2.5 – 14.6 |  |
| No information, no. (%) | 24 (6) | 4 (3) | 20 (8) |  |
| Platelets at diagnosis x10^9^/L |  |  |  | 0.04 |
| Median | 62 | 44.5 | 65.5 |  |
| Range | 2 – 916 | 2 – 407 | 4.2 – 916 |  |
| No information, no. (%) | 61(16) | 19 (15) | 42 (17) |  |
| *TP53* |  |  |  | 0.22 |
| Mutated – no. (%) | 4 (1) | 3 (3) | 1 (1) |  |
| Wildtype – no. (%) | 367 (99) | 122 (97) | 245 (99) |  |
| CR/CRi – no. (%) | 278 (75) | 88 (70) | 190 (78) | 0.19 |

Abbreviations: CR, complete remission; CRi, complete remission with incomplete hematological recovery, ECOG PS, Eastern Cooperative Oncology Group performance status; Hgb, hemoglobin; WBC, white blood cell count

**Supplementary Table S7**: Characteristics of *FLT3*-ITD^pos^/*NPM1*^mut^ patients by MRG mutation status.

| **Characteristic** | **All (n=491)** | **MRG mutation (n=46)** | **No MRG mutation**  **(n=445)** | **p** |
| --- | --- | --- | --- | --- |
| Age at diagnosis (years) |  |  |  | 0.02 |
| Median | 52 | 56.19 | 52 |  |
| Range | 18.83 – 75 | 23 – 75 | 18 .83 – 75 |  |
| Patient sex |  |  |  | 0.47 |
| Female – no. (%) | 307 (63) | 26 (57) | 281 (63) |  |
| Male – no. (%) | 184 (37) | 20 (43) | 164 (37) |  |
| ECOG PS at diagnosis |  |  |  | 0.50 |
| ECOG 0 – 1, no. (%) | 192 (39) | 16 (35) | 176 (40) |  |
| ECOG ≥ 1, no. (%) | 51 (10) | 7 (15) | 44 (10) |  |
| No information, no. (%) | 248 (50) | 23 (50) | 225 (50) |  |
| WBC at diagnosis x10^9^/L |  |  |  | 0.06 |
| Median | 45.4 | 27.4 | 46.9 |  |
| Range | 0.2 – 486 | 0.7 – 406 | 0.2 – 486 |  |
| No information – no. (%) | 25 (5) | 5 (11) | 20 (4) |  |
| Hgb at diagnosis, g/dl |  |  |  | 0.25 |
| Median | 9.0 | 9.3 | 9 |  |
| Range | 2.7 – 16 | 2.7 – 14.2 | 3.1 – 16 |  |
| No information – no. (%) | 28 (6) | 5 (11) | 23 (5) |  |
| Platelets at diagnosis x10^9^/L |  |  |  | 0.72 |
| Median | 57 | 77 | 55 |  |
| Range | 3 – 688 | 10 – 405 | 3 – 688 |  |
| No information – no. (%) | 76 (15) | 11 (24) | 65 (15) |  |
| *TP53* |  |  |  | 0.19 |
| Mutated – no. (%) | 6 (1) | 2 (4) | 4 (1) |  |
| Wildtype – no. (%) | 485 (99) | 44 (96) | 441 (99) |  |
| CR/CRi – no. (%) | 413 (84) | 39 (85) | 374 (84) | 1 |

Abbreviations: CR, complete remission; CRi, complete remission with incomplete hematological recovery, ECOG PS, Eastern Cooperative Oncology Group performance status; Hgb, hemoglobin; WBC, white blood cell count

**Supplementary Table S8:** Multivariable analysis of relapse-free survival and overall survival in *FLT3*-ITD^pos^/*NPM1*^mut^ patients (n=491).

|  | **Univariate** | | | | **Multivariable** | | | |
| --- | --- | --- | --- | --- | --- | --- | --- | --- |
|  | **HR*** | **95% CI LL** | **95% CI UL** | **p** | **HR^#^** | **95% CI LL** | **95% CI UL** | **p** |
| **RFS** | | | | | | | | |
| MRG mutant vs wildtype | 0.6 | 0.37 | 0.98 | 0.042 | 0.64 | 0.39 | 1.04 | 0.073 |
| WBC at diagnosis (increase of 1, log_2_, x10^9^/L) | 1.17 | 1.08 | 1.26 | <0.001 | 1.16 | 1.08 | 1.26 | <0.001 |
| Age (increase by 10) | 1.05 | 0.94 | 1.16 | 0.4 | - | - | - | - |
| *TP53* mutant vs wildtype | 2.2 | 0.7 | 6.88 | 0.2 | - | - | - | - |
| **OS** | | | | | | | | |
| MRG mutant vs wildtype | 0.79 | 0.51 | 1.22 | 0.3 | - | - | - | - |
| WBC at diagnosis (increase of 1, log_2_, x10^9^/L) | 1.17 | 1.09 | 1.25 | <0.001 | 1.15 | 1.07 | 1.23 | <0.001 |
| Age (increase by 10) | 1.19 | 1.08 | 1.31 | <0.001 | 1.17 | 1.06 | 1.29 | 0.002 |
| *TP53* mutant vs wildtype | 2.88 | 1.28 | 6.49 | 0.01 | 2.22 | 0.98 | 5.02 | 0.055 |

^*^Hazard ratios greater than or less than 1 indicate an increased or decreased risk, respectively, of an event for the first category listed.

Abbreviations: CI, confidence interval; CR, complete remission; LL, lower limit; alloHCT, allogeneic hematopoietic cell transplantation; WBC, white blood cell count; UL, upper limit

**Supplementary Table S9:** Univariate and multivariable analysis of overall survival considering allelic ratio of *FLT3*-ITD.

| ***NPM1*^wt^, MRG^wt^** | **Univariate** | | | | **Multivariable** | | | |
| --- | --- | --- | --- | --- | --- | --- | --- | --- |
| **RFS** | **HR*** | **95% CI LL** | **95% CI UL** | **p** | **HR^#^** | **95% CI LL** | **95% CI UL** | **p** |
| *FLT*3-ITD Allelic ratio, ≥0.05 vs < 0.05 | 3.04 | 2.04 | 4.53 | <0.001 | 2.92 | 1.95 | 4.37 | <0.001 |
| WBC at diagnosis (increase of 1, log_2_, x10^9^/L) | 1.12 | 1.02 | 1.23 | 0.019 | 1.08 | 0.98 | 1.18 | 0.11 |
| Age (increase by 10) | 1.12 | 0.97 | 1.28 | 0.11 | - | - | - | - |
| **OS** | **HR*** | **95% CI LL** | **95% CI UL** | **p** | **HR^#^** | **95% CI LL** | **95% CI UL** | **p** |
| *FLT*3-ITD Allelic ratio, ≥0.05 vs < 0.05 | 1.87 | 1.33 | 2.63 | <0.001 | 1.75 | 1.24 | 2.48 | 0.002 |
| WBC at diagnosis (increase of 1, log_2_, x10^9^/L) | 1.11 | 1.02 | 1.21 | 0.014 | 1.08 | 1.00 | 1.18 | 0.063 |
| Age (increase by 10) | 1.16 | 1.03 | 1.31 | 0.013 | 1.17 | 1.04 | 1.31 | 0.010 |

| ***NPM1*^wt^, MRG^mut^** | **Univariate** | | | | **Multivariable** | | | |
| --- | --- | --- | --- | --- | --- | --- | --- | --- |
| **RFS** | **HR** | **95% CI LL** | **95% CI UL** | **p** | **HR** | **95% CI LL** | **95% CI UL** | **p** |
| *FLT*3-ITD allelic ratio, ≥0.05 vs < 0.05 | 1.32 | 0.81 | 2.51 | 0.3 | - | - | - | - |
| WBC at diagnosis (increase of 1, log_2_, x10^9^/L) | 1.12 | 0.99 | 1.26 | 0.072 | 1.14 | 1.03 | 1.25 | 0.008 |
| Age (increase by 10) | 1.18 | 0.99 | 1.42 | 0.072 | 1.13 | 0.99 | 1.30 | 0.074 |
| *TP53* mutant vs wildtype | 2.76 | 0.37 | 20.4 | 0.3 | - | - | - | - |
| **OS** | **HR^#^** | **95% CI LL** | **95% CI UL** | **p** | **HR^#^** | **95% CI LL** | **95% CI UL** | **p** |
| *FLT*3-ITD allelic ratio, ≥0.05 vs < 0.05 | 1.26 | 0.84 | 1.89 | 0.3 | - | - | - | - |
| WBC at diagnosis (increase of 1, log_2_, x10^9^/L) | 1.14 | 1.03 | 1.26 | 0.01 | 1.20 | 1.08 | 1.33 | <0.001 |
| Age (increase by 10 ) | 1.39 | 1.19 | 1.63 | <0.001 | 1.45 | 1.24 | 1.70 | <0.001 |
| *TP53* mutant vs wildtype | 2.60 | 0.63 | 10.8 | 0.2 | - | - | - | - |

| ***NPM1*^mut^, MRG^wt^** | **Univariate** | | | | **Multivariable** | | | |
| --- | --- | --- | --- | --- | --- | --- | --- | --- |
| **RFS** | **HR^#^** | **95% CI LL** | **95% CI UL** | **p** | **HR^#^** | **95% CI LL** | **95% CI UL** | **p** |
| *FLT*3-ITD allelic ratio, ≥0.05 vs < 0.05 | 2.15 | 1.56 | 2.96 | <0.001 | 1.91 | 1.37 | 2.66 | <0.001 |
| WBC at diagnosis (increase of 1, log_2_, x10^9^/L) | 1.18 | 1.09 | 1.28 | <0.001 | 1.13 | 1.04 | 1.23 | 0.003 |
| Age (increase by 10) | 1.04 | 0.93 | 1.15 | 0.5 | - | - | - | - |
| *TP53* mutant vs wildtype | 4.23 | 0.59 | 30.5 | 0.2 | - | - | - | - |
| **OS** | **HR^#^** | **95% CI LL** | **95% CI UL** | **p** | **HR^#^** | **95% CI LL** | **95% CI UL** | **p** |
| *FLT*3-ITD allelic ratio, ≥0.05 vs < 0.05 | 1.33 | 0.98 | 1.81 | 0.07 | 1.12 | 0.81 | 1.54 | 0.5 |
| WBC at diagnosis (increase of 1, log_2_, x10^9^/L) | 1.15 | 1.07 | 1.24 | <0.001 | 1.13 | 1.05 | 1.23 | 0.001 |
| Age (increase by 10) | 1.18 | 1.07 | 1.31 | 0.001 | 1.16 | 1.05 | 1.29 | 0.005 |
| *TP53* mutant vs wildtype | 3.48 | 1.29 | 9.36 | 0.014 | 3.07 | 1.14 | 8.29 | 0.027 |

| ***NPM1*^mut^, MRG^mut^** | **Univariate** | | | | **Multivariable** | | | |
| --- | --- | --- | --- | --- | --- | --- | --- | --- |
| **RFS** | **HR^#^** | **95% CI LL** | **95% CI UL** | **p** | **HR^#^** | **95% CI LL** | **95% CI UL** | **p** |
| *FLT*3-ITD allelic ratio, ≥0.05 vs < 0.05 | 3.66 | 1.18 | 11.3 | 0.024 | 3.55 | 1.14 | 11.1 | 0.029 |
| WBC at diagnosis (increase of 1, log_2_, x10^9^/L) | 1.10 | 0.88 | 1.37 | 0.4 | - | - | - | - |
| Age (increase by 10) | 1.5 | 0.98 | 2.31 | 0.063 | 1.49 | 0.96 | 2.32 | 0.077 |
| *TP53* mutant vs wildtype | 3.19 | 0.70 | 14.5 | 0.13 | - | - | - | - |
| **OS** | **HR^#^** | **95% CI LL** | **95% CI UL** | **p** | **HR^#^** | **95% CI LL** | **95% CI UL** | **p** |
| *FLT*3-ITD allelic ratio, ≥0.05 vs < 0.05 | 3.77 | 1.27 | 11.2 | 0.017 | 3.58 | 1.20 | 10.7 | 0.022 |
| WBC at diagnosis (increase of 1, log_2_, x10^9^/L) | 1.24 | 1.01 | 1.52 | 0.04 | 1.12 | 0.98 | 1.48 | 0.072 |
| Age (increase by 10) | 1.54 | 1.03 | 2.29 | 0.033 | 1.49 | 0.99 | 2.23 | 0.056 |
| *TP53* mutant vs wildtype | 2.75 | 0.63 | 12.1 | 0.2 | - | - | - | - |

Abbreviations: CI, confidence interval; CR, complete remission; LL, lower limit; WBC, white blood cell count; UL, upper limit

**Supplementary Figure S1**

**Supplementary Figure S1:** Patient selection from the HARMONY Alliance consortium database, starting from adult AML patients who were treated with intensive chemotherapy and for whom genetic data was available on mutational status of *FLT3, NPM1* and all nine MRG mutation.

**Supplementary Figure S2**


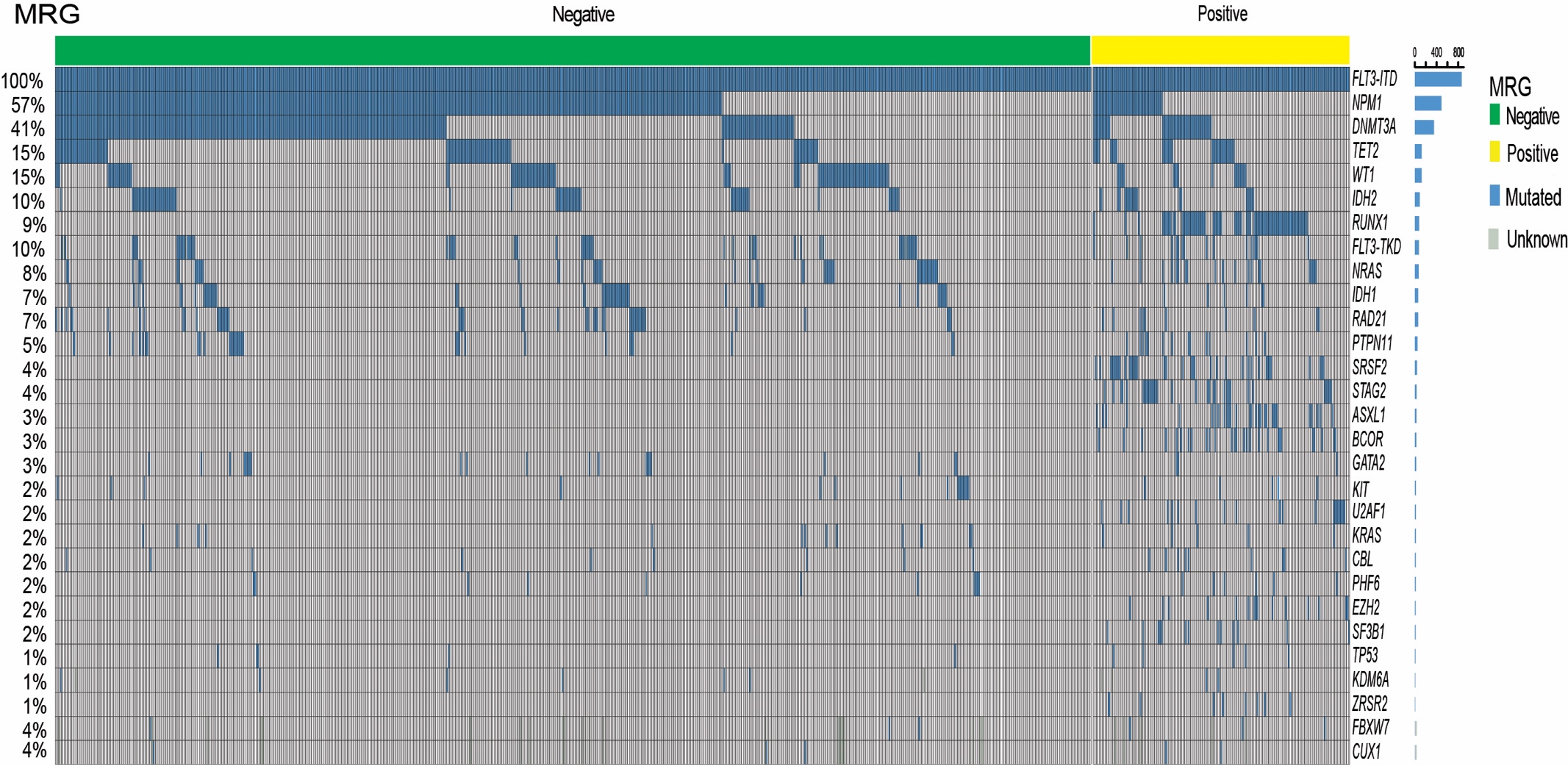


**Supplementary Figure S2: Oncoprint depicting the mutational landscape of *FLT3*-ITD positive AML.** The patients are ordered by the presence (yellow) or absence (green) of MRG mutations.

**Supplementary Figure S3**

**
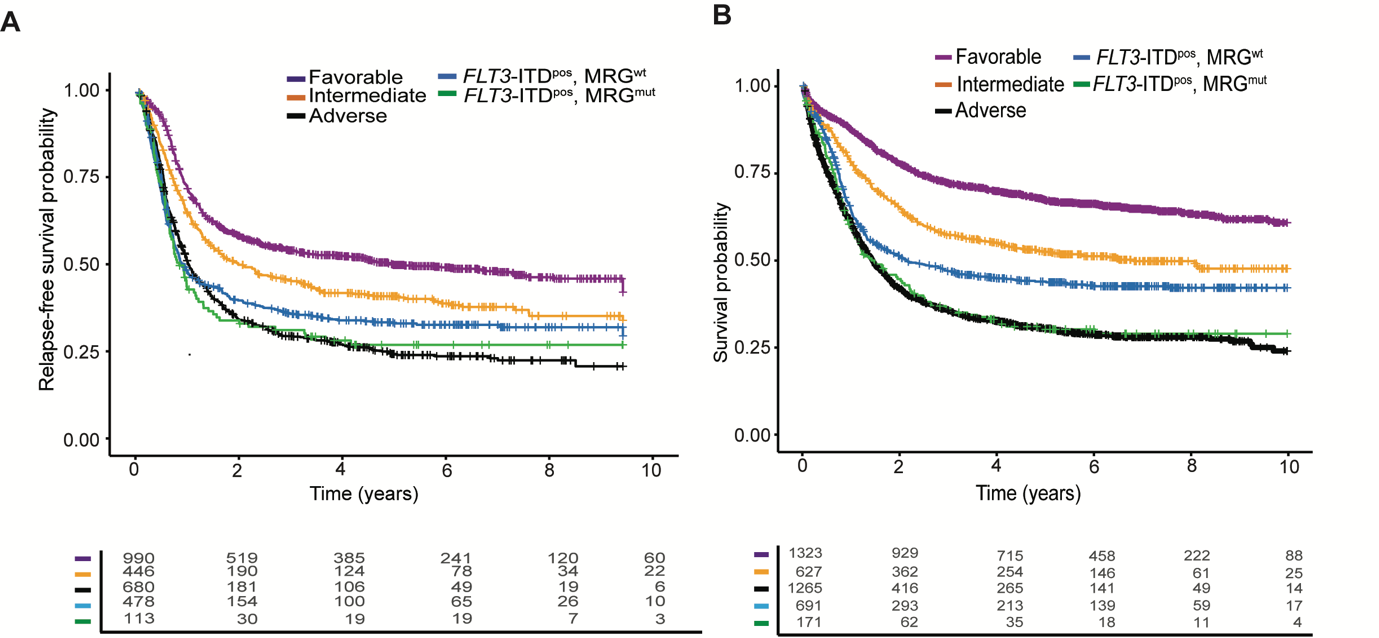
**

**Supplementary Figure S3: Outcome of *FLT3*-ITD^pos^ patients compared to ELN risk categories.**

RFS (A) and OS (B) of *FLT3*-ITD^pos^ patients with and without MRG co-mutations in comparison to the three ELN 2022 risk groups.

**Supplementary Figure S4**

**
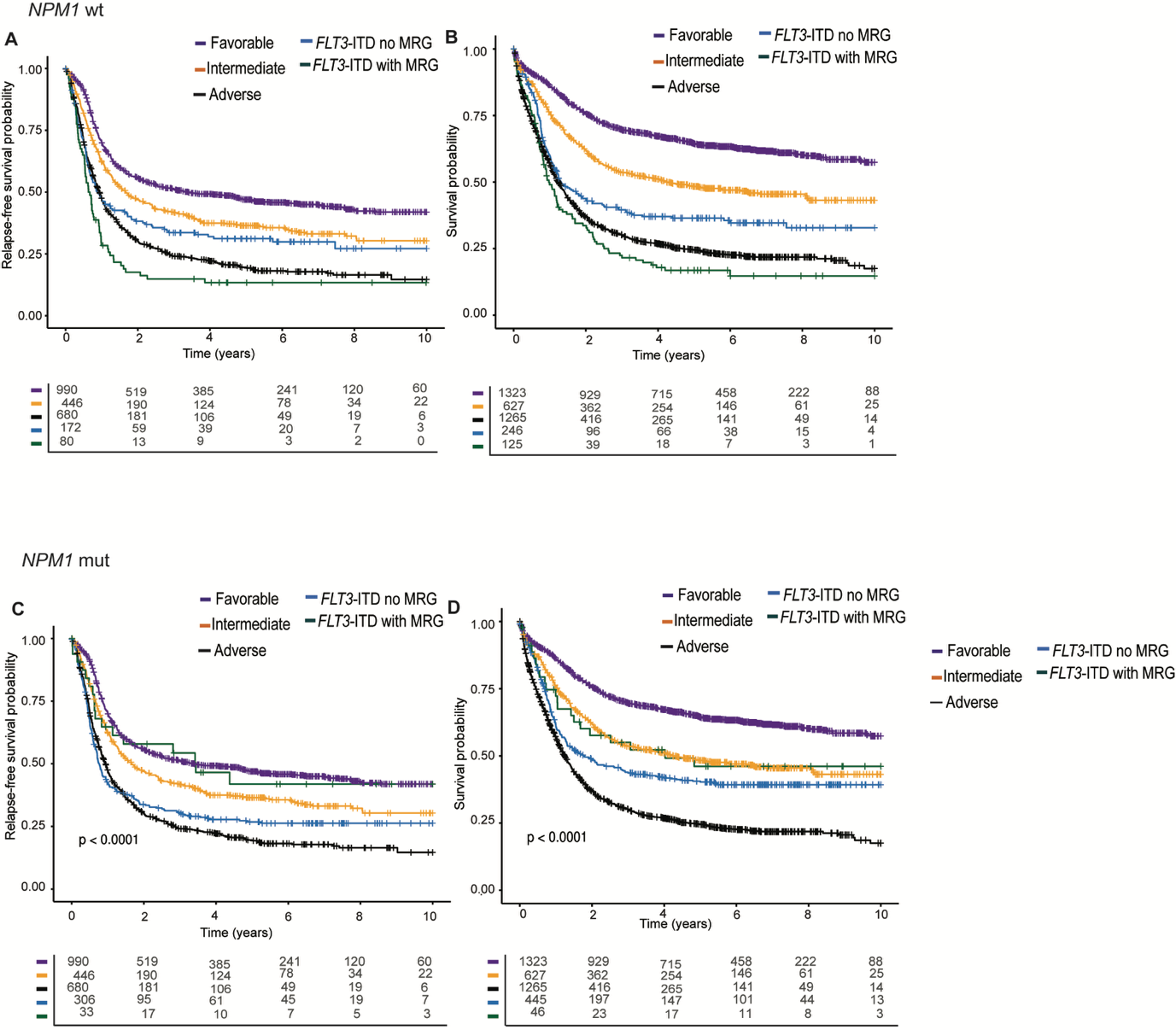
**

**Supplementary Figure S4:** **Outcome of *FLT3*-ITD patients compared to ELN risk categories stratified by *NPM1* mutational status.**

RFS (left) and OS (right) of *FLT3*-ITD positive patients without (A and B) and with (C and D) *NPM1* co-mutations stratified by the presence of MRG mutations in comparison to the ELN 2022 risk groups.

**Supplementary Figure S5**

**
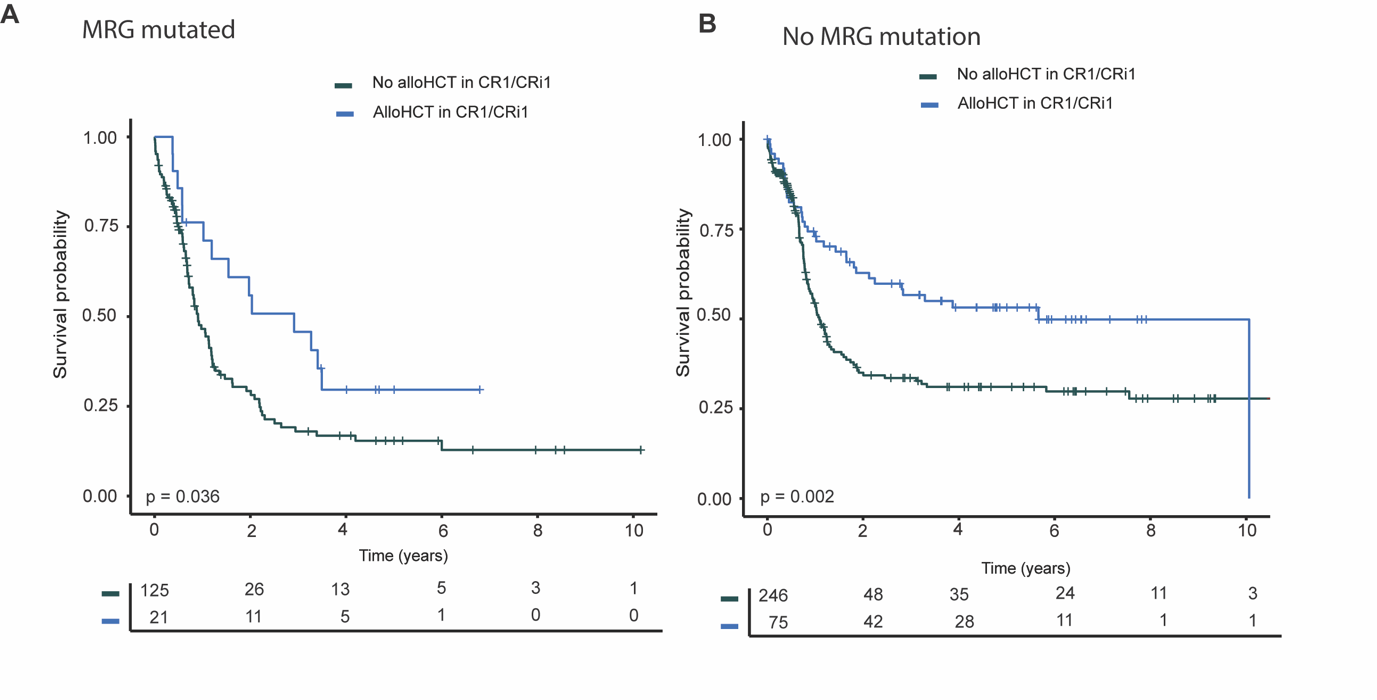
**

**Supplementary Figure S5: Analysis for OS in *FLT3*-ITD^pos^/*NPM1*^wt^ patients according Simon Makuch.**

OS (A) in *FLT3*-ITD^pos^/*NPM1*^wt^ patients with MRG mutation undergoing or not undergoing alloHCT in first CR/CRi.

OS (B) in *FLT3*-ITD^pos^/*NPM1*^wt^ patients without MRG mutation undergoing or not undergoing alloHCT in first CR/CRi.

**Supplementary Figure S6**
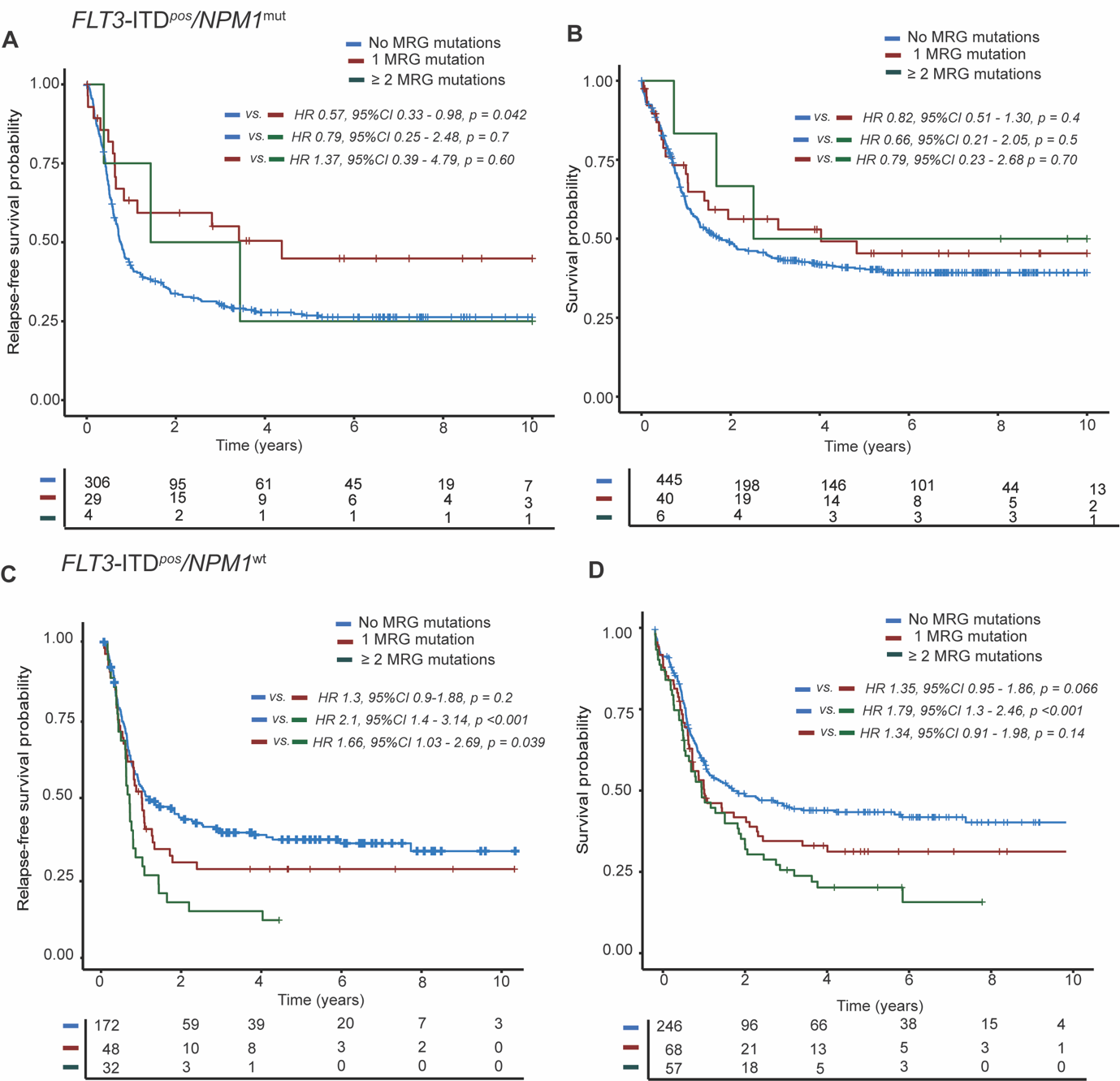


**Supplementary Figure S6. Prognostic impact of the number of MRG mutations per patient.**

RFS (A) and OS (B) of patients with MRG mutations stratified by the presence of 1 or ≥2 MRG mutations compared to patients without MRG mutation within *FLT3*-ITD^pos^/*NPM1*^mut^ patients. RFS (C) and OS (D) of patients with MRG mutations stratified by the presence of 1 or ≥2 MRG mutations compared to patients without MRG mutation within *FLT3*-ITD^pos^/*NPM1*^wt^ patients.

**Supplementary Figure S7
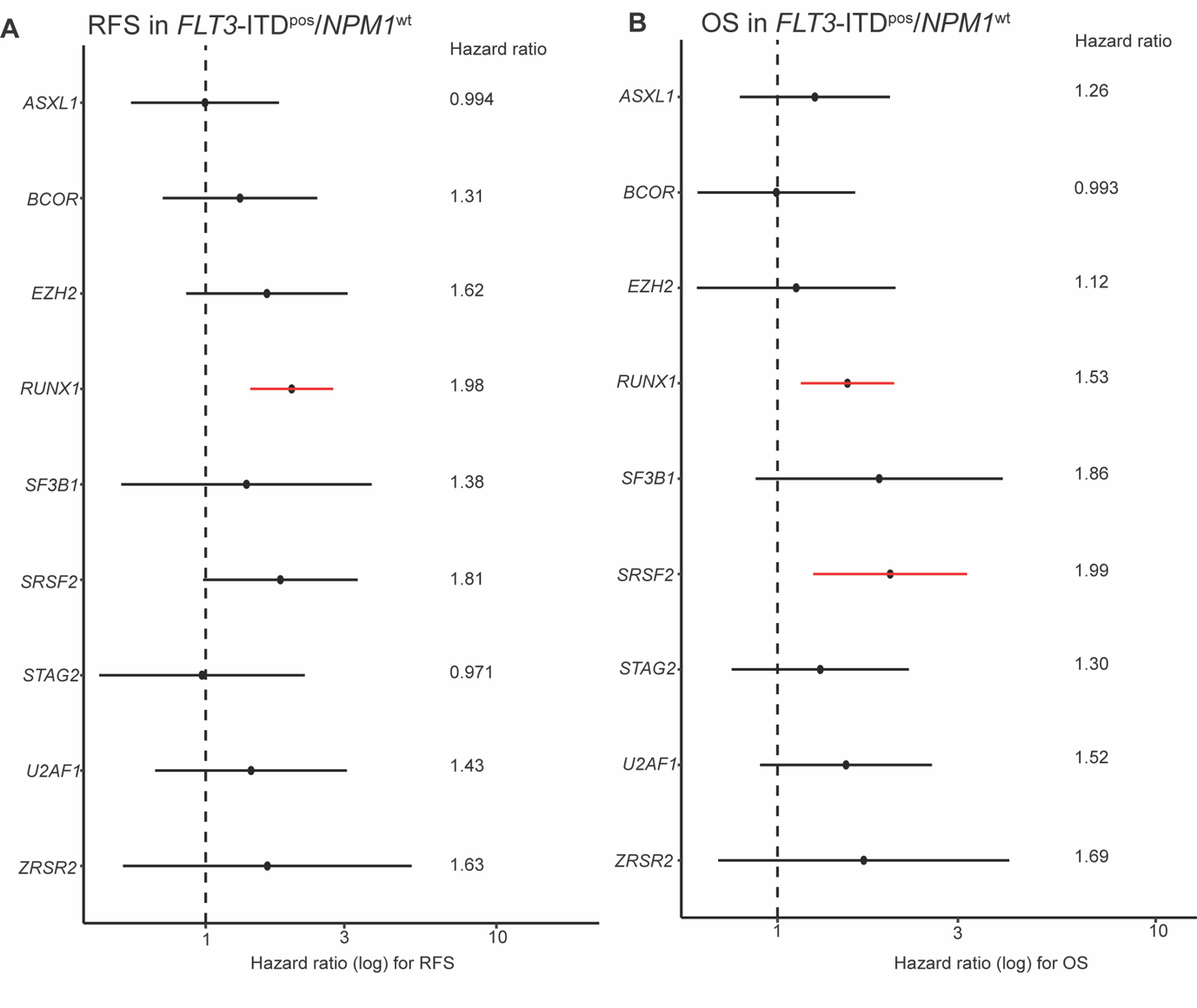
**

**Supplementary Figure S7: Prognostic impact of individual MRG mutations in *FLT3*-ITD^pos^/*NPM1*^wt^ patients.**

Forest plot depicting the impact of individual MRG mutations on RFS(A) and OS (B) in *FLT3*-ITD^pos^/*NPM1*^wt^ patients.
